# The effectiveness of mindfulness-based cognitive therapy on quality of life and loneliness of women with HIV

**Published:** 2015

**Authors:** E Samhkaniyan, A Mahdavi, S Mohamadpour, S Rahmani

**Affiliations:** * PhD of Student Health Psychology, department of education and psychology, Islamic Azad University, Karaj, Iran; ** Master of Clinical Psychology, department of education and psychology, Islamic Azad University, Science and Research Branch, Ahvaz, Iran; *** Master of Clinical Psychology, Social Determinants of Health Research Center, Lorestan University of Medical Sciences, Khorramabad, Iran; **** PhD of Student Health Psychology, Azad University Islamic, Karaj, Iran. Behavioral Sciences Research Center of Shahid Beheshti University of Medical Sciences

**Keywords:** positive HIV woman, quality of life, loneliness, mindfulness

## Abstract

**Objective:** The present study investigated the performance of Mindfulness according to the Cognitive approach on the Quality of Life and Loneliness of women with HIV.

**Methods:** This research is a semi-experimental with pretest-posttest and check team, which was conducted in winter, 2014. In this research, 24 positive HIV women in Tehran were selected by volunteers sampling method and were stochastically related to either the control team (n = 12) or the MBCT groups (n = 12) and, the World Health Organization quality of life survey and the University of California Los Angeles loneliness scale were administrated as pretest. The MBCT team got eight sessions of mindfulness according to the cognitive theory and the check team got no intervention. At the end, the post-test was administrated to two groups and, covariance method was used for data analysis by SPSS-20 software.

**Findings:** The results of the present study indicated that there were clear variations among the test groups check group and MBCT (p < 0.001). Therefore, Mindfulness-based Cognitive theory increased the mean quality of life and decreased loneliness.

**Conclusion:** The findings indicated that the Mindfulness-based Cognitive therapy increased the quality of life and decreased loneliness in positive HIV women. Therefore, in order to modify the quality of life and loneliness in these cases, attention to these variables during clinical trials with the goal of an appropriate intervention, will be beneficial.

## Introduction

Lately, HIV infection and AIDS have been among the world’s most common infectious diseases, which have been associated with the high burden of the disease for the patients [**1**], so that it was estimated that since 2012, about 35 million persons have been living with the HIV virus around the world, and approximately 3.2 million people have been newly infected [**2**]. Human immunodeficiency virus (HIV) is among the retroviral infections which targets the immune system and with the advancement and proliferation of the virus, patients will be affected by the acquired immune deficiency syndrome (AIDS) [**1**]. Since the antiretroviral treatment (ART) was introduced and developed, the access and use of medical care for HIV-positive people has increased. Therefore, these people were able to benefit from a longer life and their status of being infected with a deadly disease has changed to a manageable disease [**2**]. The treatment that the people with HIV receive requires a reconsideration of their psychological care. In addition, in the past, it was merely trying to save the patient’s death but today, with the advances in the treatment, the life expectancy of patients has increased and it is expected to modify their health-associated quality of life [**3**].

Canker and NATO (1995) [**3**] have defined the health-associated quality of life as being a subjective assessment of the cases regarding the effect of their health status, the received fitness administration and health promotion actions on the capability of reaching and keeping a satisfactory stage of performance, which allows people pursue their life objectives and reflects their general well-being. In other words, health-related quality of life is not limited only to factors associated with the progression of HIV, but psychological factors have a decisive role in defining it.

In fact, the risk of being affected by the HIV virus is a complex phenomenon, followed by many factors, including depression, social isolation, discrimination, poverty and suffering symptoms of the disease [**4**], the length of the diagnosis, sorrows [**5**], stigma, loss of social support, the burden of multiple symptoms of HIV, physical and psychological diseases at the same time, and the development of personality disorders, which are very effective in reducing the quality of life [**6**]. In addition, several studies have shown that many of these factors are more severe in women with HIV [**7**]. Therefore, conducting the research on factors that increase the quality of life of these patients would be very important.

Also, one of the major themes of life in patients with HIV, that is one of the main sources of pressure and distress in these patients, is loneliness [**4**]. Loneliness has been described as the difference between current and ideal situations in achieving communication with others and society [**8**]. Previous studies have been conducted on loneliness in patients with HIV. For example, Stanton et al. [**4**] investigated the loneliness in HIV patients and showed that feeling alone in these people leads to behaviors such as smoking that intensifies their illness. Also, Randolph et al. [**8**] showed in their study that loneliness was more related to the stigma and to the risky sexual behavior in these individuals.

Despite doing a lot of research about the loneliness of patients with HIV and factors associated with it, while reviewing the research background about the studies conducted in Iran, it was found that there was no study examining the effectiveness of psychological treatments on the loneliness of these people. Therefore, there is a gap in this field, which led to the necessity of conducting such a research. This is despite the fact that numerous studies have shown that mindfulness techniques have led to an improvement of self-management regarding the issues related to the health of people with HIV [**5**]. Mindfulness means paying attention in a particular goal-oriented manner at the present moment without judgment and prejudice [**9**].

In the mindfulness treatment, people are encouraged to pay attention to their inner experiences at any moment, physical sensations, thoughts, and feelings and also to the environmental aspects such as sights and sounds. During this treatment, it is recommended that the attitude of accepting the affairs without judgment should be created in these people, which means the creation of awareness in people about the perceptions cognitions, emotions or feelings but without judgment and evaluation about the good or bad, true or false, being important or not, being healthy or not (Khoury et al., 2011) [**10**].

The Mindfulness Based Cognitive Therapy was first used in order to prevent depression. However, it was observed that this treatment improves the clinical disease, quality of life, and the emotional status in various clinical diseases such as cancer and cardiovascular diseases [**5**].

Mindfulness-based cognitive therapy is a combination of elements and principles of mindfulness along with cognitive therapy whose effectiveness was used in several studies to reduce the side effects of anti-retroviral treatments [**11**], improving the quality of life, and improving the management of the emotional states [**5**] in patients with HIV.

However, there has been no study in this area in Iran. According to what was mentioned and the difficulty in the clinical management of people with HIV, the psycho educational interventions (non-drug) could be a good therapeutic option for reducing their psychological discomfort [**5**]. However, it seems that the necessity of paying attention to this issue is more prominent in women. Because, in spite of very effective treatments to control HIV, the mortality rates in women with HIV is alarmingly high and this shows the importance of paying more attention to them [**12**]. Thus, in order to address this research gap and the need to modify the quality of life in these cases, it is necessary to conduct such a study. Therefore, the present study examined and discussed the performance of mindfulness-based cognitive theory on the quality of life quality and loneliness among the females with HIV.

## Research Method

The study was based on the quasi-experimental model with pretest-posttest and check team. The investigation population included all women in Tehran (the capital of Iran) who were positive at the period of the study, among which a sample of 24 patients who had inclusion and exclusion criteria for the study and were satisfied to participate were selected. The inclusion criteria included the consent and informative willingness to participate in the research, the ability to participate in meetings and collaborate on assignments, to work in completing the instruments, the physical and psychological stability (lack of intervening physical or psychological symptoms during sessions, the treatment including fatigue, muscle aches, etc.).

The minimum education was the pre-high school degree and the age range of the participants was 20 to 45 years.

Also, the patient was excluded if she was treated because of a physical or psychological illness or in the presence of cognitive impairment or impaired cognitive function, acute and severe symptoms so that the patients’ participation in the present was difficult or almost impossible. The procedure of the study was that, for assessing the efficiency of mindfulness-based cognitive approach on the quality of life and loneliness of the women via HIV, the pretest-posttest design with the control group was used, which included the following stages of implementation.

From the patients who had volunteered for the study, a total of 24 patients with the inclusion and exclusion criteria were selected. Then, the letter A was written on 12 sheets, and the letter B on 12 sheets, totally 24 sheets being prepared. During the pre-test, the World Health Organization Life Quality Survey and the revised UCLA Loneliness Scale of Russell (UCLA) were provided for each patient individually by the researcher and each patient was asked to choose 1 sheet from the prepared sheets. This way, all the cases were stochastically classified to 2 teams of A and B and then they were stochastically related to the check team and MBCT.

Therefore, the experimental group took the MBCT for 8 sessions each seven day, a session lasting for 2 hours and the control group received no intervention. At the end of the practicing sessions, the cases took part in post-test. The Mindfulness-based cognitive MBCT is presented in **Table 1**.

**Table 1:** Functional guidance sessions of MBCT

Session	Topic
First session: Automatic Pilot	The initiation of automatic, intuitive system/ knowing how to use current moment practice of physic sensation, feelings and mental in decreasing anxiety/ training lowering raisins (Object recognition practicing), providing feedback and conversation regarding the practice/ 3-minutes breathing, giving assignment for the next 7 days and spreading pamphlets of the initial session and CDs of meditation
Second session: facing obstacles	Re-examining body workout/ providing response and conversation regarding the examination of body workout/ training breathing mindfulness reflection/ spreading leaflets of the time session and CDs of thought
Third session: Kindness with breathing, body and awareness about breathing and body movement	Having conscious sitting with awareness of breathing (the relaxing study)/ training 3-minutes breathing/ spreading leaflets of the 3rd session and videotape of yoga exercises
Fourth session: learning how to answer	Re-examining the body workout/ (in the infirmary, oratory)/ five-minutes functioning of “looking or understanding”/ re-training known session with knowledge of living and body/ spreading leaflets of the 4th session and CDs of thought
Fifth session: slowly cope with difficulty (attendance)	Training living/ re-training known session (knowledge of breathing, physic, notes and images)/ defining the pressure and specifying cases’ responses to anxiety/ evaluating knowledge of comfortable and offensive issues on responding, feelings, and physical sensations/ training 3-minutes breathing / distributing leaflets
Sixth session: thoughts are not facts	Training relaxing meditation (mindfulness of notes and ideas)/ spreading leaflets of the sixth session and number 4 videotape to members
Seventh session: self-care	Training mountain meditation/ sleep hygiene/ recurring activities of the former session/ making a list of pleasant actions/ spreading leaflets of the 7th session
8 session: going beyond fear	Investigating physic work/ overview of plans/ reviewing and consulting arrangements/ training stone, beads and toys thought

The instruments used in this study included the samples of the demographic information sheet, the World Health Organization Life Quality Survey and The revised UCLA Loneliness Scale of Russell (UCLA), respectively.

**Research Instruments**

**The sample sheet of the demographic information**

This sample sheet included the data regarding age, and education level, which was developed and evaluated by the researcher of the present study. 

**The World Health Organization Quality of Life Questionnaire **

This survey was prepared via the World Health Organization (1996) in order to investigate the quality of life and it is widely used. The short form of this survey contained 26 questions and assessed 4 regions of the body health (seven index), emotional care (6 questions), community relationships (3 questions) and environment (8 questions) with 24 questions regarding the degree and 2 questions which also assessed the total quality of life.

The range of the scores in each area was between 4 (worst condition) to 20 (best condition) [**13**]. In Iran, Nejat et al. [**14**] evaluated the scale standardization and obtained the alpha coefficient of the questionnaire for a healthy people within the body care of 0.7. 

Mental care was of 0.73, social relationships of 0.55 and environment relations of 0.84. Moreover, after two weeks, they reported the retest reliability coefficient of 0.7. 

The study of Nasiri and Joukar (2008) [**13**] of 26 items of the scale factor analysis, revealed that the following four main domains of the scale such as body health, social links, psychological care, and social condition on this scale, represent the construct validity of the questionnaire. Also, Cronbach’s alpha factor of 0.84 was obtained for the questionnaire. 

**The revised UCLA Loneliness Scale of Russell (UCLA)**

The revised UCLA Loneliness Scale was developed by Russell, Peplauand Cortana (1980) and had 20 questions with four points Likert rate from never (score 1) to always (score 4), out of which 10 questions were positive and 10 questions were negative. The individual score was obtained from the sum of the 20 questions and the range of scores could be between 20 and 80. There was the possibility of bias in responses given to the UCLA-scale. For this purpose, the experts decided that the new scale should be corrected [**15**]. This scale was translated by Shokrkon and Mirdrikvand (1998) [**16**] and was used after a pilot and primary implication.

Russell and Ferguson developed the revised UCLA Loneliness Scale of Russell (UCLA) for the first time and after a three times edition, the final version of the scale was conducted in four groups of students, nurses, teachers and the elderly in different forms such as self-reports and interviews. The alpha coefficient ranged between 0.89 and 0.94. The test was conducted a year later among the elderly people and the test-retest correlation of 0.73 was obtained, which was satisfactory [**15**].

## Results

The average and nominal deviation of the cases age in this study was 54/ 5 ± 38/ 36 respectively. In terms of marital status, 4 participants were single (16.7 percent), 14 were married (3/ 58) and 6 (25%) were divorced. In terms of education, 7 participants (29.2%) were low literate, 15 participants (5/ 62 percent) had a high school diploma and 2 patients (3.8%) had a college degree. In The average and nominal deviation of the quality of life and loneliness in women via HIV are provided in **Table 2**.

**Table 2:** The average and nominal deviation of the quality of life and loneliness in women with HIV

Variable			Experimental (n = 12) Average ± Nm		Check (n = 12) Average ± Nm	
			Pre-test	Post-test	Pre-test	Post-test
Quality of life	Dimension	Physical	11.12 ± 1.31	13.92 ± 1.53	11.33 ± 1.10	11.18 ± 1.07
		mental health	10.67 ± 0.77	12.76 ± 0.89	10.21 ± 0.66	10.01 ± 0.65
		Social	9.80 ± 0.95	11.94 ± 1.31	10.05 ± 0.91	9.96 ± 0.93
		Environmental health	9.91 ± 0.51	11.60 ± 0.82	10.75 ± 1.02	10.58 ± 1.22
		Total score	41.51 ± 1.75	50.25 ± 1.78	42.34 ± 1.76	41.74 ± 1.74
		Loneliness	43.58 ± 6.78	55.17 ± 8.16	44.33 ± 6.22	44.83 ± 6.13

As it can be seen in **Table 3**, the quality of life scores in the test team increased from pre to post tests in all dimensions (physical, mental health, social and environmental). The greatest increase was seen in the physical domain. The total score quality of life of patients with HIV increased from pre-test to post-test, which was also the sign of improvement in the quality of life. Also, the mean and standard deviation of the pre-test to post-test loneliness were also declined.

In this research, the statistical analysis of the quality of life and loneliness scores in the two tests and control groups were performed with a univariate analysis of covariance and multivariate analysis of covariance. Prior to the analysis, the assumptions of the model were investigated. **Table 3** shows the results of the tests of Levene’s test regarding the evaluation of the consistency of the variances in the posttest and in **Table 4**. Multivariate analysis of covariance (QOL) and univariate analysis of covariance are presented in the control pre-test and post-test scores on the quality of life and loneliness variables.

**Table 3:** The results of the tests of Levene’s test regarding the evaluation of the consistency of the variances in the posttest

Modality	Dimension	F	Df1	Df2	significance level
Quality of life	Physical	1.317	1	22	0.263
	mental health	2.058	1	22	0.165
	Social	1.229	1	22	0.267
	Environmental health	2.152	1	22	0.157
Total score		0.001	1	22	0.974
Loneliness		0.184	1	22	0.672

As it can be seen in **Table 3**, the proposal of the variances equality in both stages is verified. The variances of the 2 teams are identical in community and have no clear variation. Thus, according to the assumption of the Levene’s test, the possibility of implementing the results of covariance analysis to investigate the hypothesis is permitted. 

**Table 4:** Multivariate analysis of covariance (QOL) and univariate investigation of covariance via the control pre-test to post-test rates on the quality of life

Exam name	amount	F	Df	notable level	Eta square	potency
Pylayy trace test	0.938	72.161	4	0.001	0.938	0.95
Wilks Lambedai test	0.062	72.161	4	0.001	0.938	0.95
Hotelling effect test	15.192	72.161	4	0.001	0.938	0.95
Largest root test	15.192	72.161	4	0.001	0.938	0.95

As it can be observed in **Table 4**, the notable stage of all the tests (p < 0.001) indicating that there is a clear distinction between the 2 groups was at least in one of the dependent parameters and on the basis of Chi Eta, 0.93% of the differences observed in the variable of the quality of life among individuals was related to the impact of the independent variable, that being the intervention. On the other hand, given that the statistical power was 0.95 and higher than 0.80, the sample size for this study was acceptable.

The results related to significant differences in any of the dependent variables are listed below.

**Table 5:** Results of multi-parameter and univariate investigation of covariance regarding the evaluation of the efficiency of MBCT on the quality of life and loneliness in the post-test

Index	Sum of squares	dF	Mean squares	F	Notable level	Eta square
Physical	45.128	1	45.128	25.626	0.001	0.538
mental health	45.927	1	45.927	75.170	0.001	0.774
Social	23.207	1	23.207	18.016	0.001	0.450
Environmental health	6.335	1	6.335	5.794	0.025	0.208
Global Quality of life	433.840	1	433.840	138.053	0.001	0.863
Loneliness	640.667	1	640.667	0.358	0.002	.0358

As presented in **Table 5**, the notable stage was lower than 0.05 variables (p < 0.05). Therefore, the hypothesis of the difference between the quality of life and loneliness in the post-test between the experimental and control groups was confirmed. According to the ETA coefficient, 53 percent of the change in the physical dimension, 77 percent change in the mental health score, 45 percent change in the social aspect and 20 percent change in the environmental health score, 86 percent change in the overall quality of life score and 35 percent change in the loneliness variable score, were due to the independent variable (MBCT).

Also, according to the results presented in **Table 2** it could be concluded that MBCT improves the quality of life and reduces the loneliness of cases who suffer from HIV.

## Discussion and Conclusion

The purpose of this research was to evaluate the efficiency of MBCT on the quality of life and loneliness in women with HIV and the findings of investigation of covariance indicated that it had a clear positive impact on the quality of life of cases suffering from HIV. 

The findings were in line with the study of the Gonzalez-Garcia et al. [**5**], who investigated the MBCT, which examines the quality of life in patients with HIV and showed that this intervention has been imperative in increasing the quality of life of these cases. Moreover, these findings were in line with the studies of Godfrey et al. [**17**], Kaviani et al. [**18**], Akbariet et al. [**19**] and Rahmani et al. [**20**]. While trying to explain the findings, Gonzalez-Garcia et al. [**5**] suggested that two ways mindfulness-based therapies could lead to the increase of the quality of life of cases suffering from HIV.

The first is that mindfulness can directly increase the quality of life of cases suffering from HIV through changes in patient behavior, such as diet, smoking, medication adherence, etc. Also, by decreasing the negative psychological signs in patients with HIV such as stress, depression and anxiety, MBCT leads to an increase in the quality of life in patients with HIV.

Masoomian et al. [**20**] also explained the similar findings in relation to patients with chronic pain, stating that mindfulness with reduced response to distressing thoughts and feelings of patients have resulted in an improved psychological state and the increase in their physical self-monitoring and awareness to the body, resulted in improved physical mechanisms and caring. In addition, similarly to the traditional relaxation training, the mindfulness meditation was associated with an increased parasympathetic activation that could lead to the deep relaxation of the muscles and reduce tension and arousal and all of these factors affect the quality of life of the people. Moreover, the findings of the investigation showed that this therapy had a significant positive impact on reducing the loneliness of the patients with HIV. No similar studies were found in Iran. However, the findings of the study were in agreement with the outcomes of Creswell [**21**] and Rousenstrechet al. [**22**]. Regarding the explanation of their similar findings, that the mindfulness-based treatment reduces the mental loneliness, Creswell et al. [**21**] stated that the mindfulness practices in different methods, such as reducing the internal disturbing factors like intrusive thoughts, provides a more relaxed area and this leads to a reduced interpersonal sensitivity in men and it can reduce feelings of loneliness.

In the explanation of mindfulness associated with a reduction in the feelings of loneliness, Rousenstrech et al. [**22**] suggested that mindfulness leads to a decrease in the loneliness through three mechanisms. It is the first mechanism through which the mindfulness practices lead to the improvement of attention, memory, executive function, and cognitive relaxation. However, loneliness is accompanied by cognitive deficits such as the lack of any commitment to relationships and leaving the relationship or the increased memory alignment for social information while mindfulness exercises can reduce the cognitive effects of the memory. The second mechanism was mindfulness, which led to reduced feelings of loneliness through signal reduction of the internal stress. In this case, mental loneliness was usually caused by stress, anxiety, and cortisol levels. Therefore, mindfulness reduces the loneliness by reducing these feelings. A third mechanism through which mindfulness seems to reduce feelings of loneliness is the change in the perception of the self. For example, loneliness is associated with low self-esteem while the practice of mindfulness challenged those feelings and weakened them.

## Conclusion

Based on the outcomes, team MBCT is a suitable and proper method in decreasing the Loneliness and increasing the quality of life in HIV women.
